# Pharmacological Inhibition of STAT6 Ameliorates Myeloid Fibroblast Activation and Alternative Macrophage Polarization in Renal Fibrosis

**DOI:** 10.3389/fimmu.2021.735014

**Published:** 2021-08-26

**Authors:** Baihai Jiao, Changlong An, Melanie Tran, Hao Du, Penghua Wang, Dong Zhou, Yanlin Wang

**Affiliations:** ^1^Division of Nephrology, Department of Medicine, University of Connecticut School of Medicine, Farmington, CT, United States; ^2^Department of Immunology, University of Connecticut School of Medicine, Farmington, CT, United States; ^3^Department of Cell Biology, University of Connecticut School of Medicine, Farmington, CT, United States; ^4^Institute for Systems Genomics, University of Connecticut School of Medicine, Farmington, CT, United States; ^5^Renal Section, Veterans Affairs Connecticut Healthcare System, West Haven, CT, United States

**Keywords:** fibroblasts, macrophages, extracellular matrix, renal fibrosis, STAT6

## Abstract

A hallmark of chronic kidney disease is renal fibrosis, which can result in progressive loss of kidney function. Currently, there is no effective therapy for renal fibrosis. Therefore, there is an urgent need to identify potential drug targets for renal fibrosis. In this study, we examined the effect of a selective STAT6 inhibitor, AS1517499, on myeloid fibroblast activation, macrophage polarization, and development of renal fibrosis in two experimental murine models. To investigate the effect of STAT6 inhibition on myeloid fibroblast activation, macrophage polarization, and kidney fibrosis, wild-type mice were subjected to unilateral ureteral obstruction or folic acid administration and treated with AS1517499. Mice treated with vehicle were used as control. At the end of experiments, kidneys were harvested for analysis of myeloid fibroblast activation, macrophage polarization, and renal fibrosis and function. Unilateral ureteral obstruction or folic acid administration induced STAT6 activation in interstitial cells of the kidney, which was significantly abolished by AS1517499 treatment. Mice treated with AS1517499 accumulated fewer myeloid fibroblasts and myofibroblasts in the kidney with ureteral obstruction or folic acid nephropathy compared with vehicle-treated mice. Moreover, AS1517499 significantly suppressed M2 macrophage polarization in the injured kidney. Furthermore, AS1517499 markedly reduced the expression levels of extracellular matrix proteins, and development of kidney fibrosis and dysfunction. These findings suggest that AS1517499 inhibits STAT6 activation, suppresses myeloid fibroblast activation, reduces M2 macrophage polarization, attenuates extracellular matrix protein production, and preserves kidney function. Therefore, targeting STAT6 with AS1517499 is a novel therapeutic approach for chronic kidney disease.

## Introduction

Chronic kidney disease (CKD) has become a significant public health challenge (1). Fibrosis is the ultimate common pathway for development of CKD and is considered a major factor for the progression of all forms of CKD (2). Renal fibrosis is a pathological feature of CKD, leading to the replacement of normal kidney tissue structure with extracellular matrix (ECM) with progressive and irreversible damage to kidney function (3). However, despite increased understanding of the molecular mechanisms of renal fibrosis, there is no specific treatment to control fibrosis and restore renal function. As the origin and functional contribution of fibroblasts are not completely understood, effectively targeting fibroblasts in organ fibrosis remains a challenge.

Accumulating evidence indicates that myeloid myofibroblasts termed fibrocytes play a crucial role in the process of fibrosis (4–7). We and others have previously shown that the recruitment of bone marrow-derived fibroblasts increases the progression and development of renal fibrosis (8–12). Therefore, targeting these cells may serve as an effective therapeutic strategy to treat chronic kidney disease. Bone marrow-derived fibroblasts share the characteristics of mesenchymal cells as well as hematopoietic cells (13, 14). However, various cytokines produced in the local microenvironment determine the differentiation of bone marrow-derived fibroblasts. We have previously demonstrated that Th2 cytokines (IL-4 and IL-13), which are considered profibrotic cytokines, enhances the expression of type I collagen, fibronectin and α-SMA in bone marrow-derived monocytes (15, 16).

STAT6 signaling is primarily activated by Th2 cytokines such as IL-4 and IL-13 and is associated with the pathogenesis of lung (17, 18), liver (19) and renal fibrosis (15, 20). We have recently shown that JAK3/STAT6 plays a crucial role in the activation of bone marrow-derived fibroblasts and development of renal fibrosis in obstructive nephropathy (15). Furthermore, we have shown that knockout of IL-4Rα inhibits STAT6 activation, myeloid fibroblast accumulation and transformation into myofibroblasts, M2 macrophage polarization, and development of renal fibrosis following obstructive injury or folic acid administration (16). These findings suggest that STAT6 signaling pathway may serve as a novel therapeutic target for renal fibrosis.

In this study, we evaluated the potential therapeutic role of AS1517499, a potent and selective STAT6 inhibitor (21), in myeloid fibroblast activation and development of renal fibrosis in two experimental murine models. Our results demonstrate that AS1517499 abolished STAT6 activation in the interstitial cells of the kidney with obstructive injury or folic acid nephropathy. Furthermore, administration of AS1517499 suppresses the activation of myeloid fibroblasts, reduces M2 macrophage polarization, and attenuate renal fibrosis. These findings suggest that inhibition of STAT6 with AS1517499 has the potential for the treatment of fibrotic kidney disease.

## Materials and Methods

### Materials

**Figure 1:**
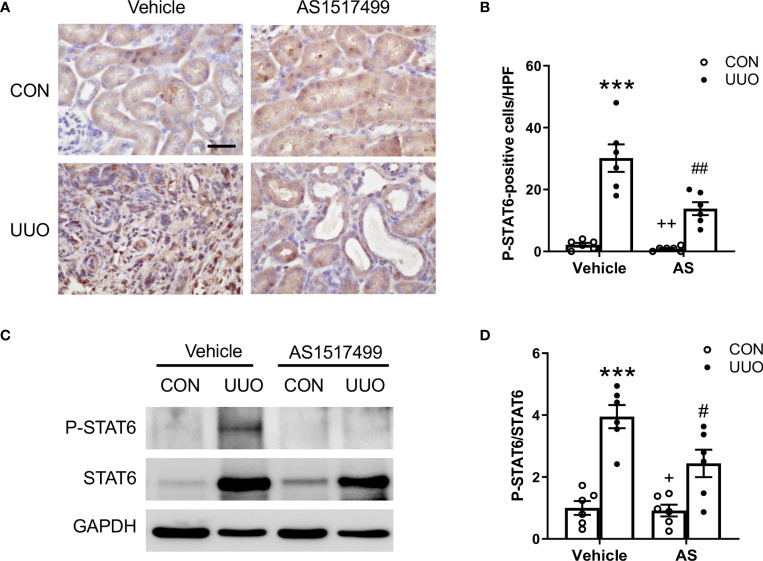
AS1517499 inhibits STAT6 activation in the kidney with obstructive injury. **(A)** Representative photomicrographs of kidney sections at day 10 of UUO stained for phosphorylated STAT6 (brown) and counterstained with hematoxylin (blue). Scale bar, 50μm. **(B)** Quantitative analysis of phosphorylated STAT6-positive cells in the kidney. ***P < 0.001 *vs*. Vehicle-CON; ^##^ *P* < 0.01 *vs* Vehicle-UUO; ^++^ *P* < 0.01 *vs* AS-UUO. n = 6 per group. **(C)** Representative Western blots show protein levels of p-STAT6 and STAT6 in the kidney at day 10 of UUO. **(D)** Quantitative analysis p-STAT6 protein levels in the kidney. ***P < 0.001 *vs*. Vehicle-CON; ^#^ *P* < 0.05 *vs* Vehicle-UUO; ^+^ *P* < 0.05 *vs* AS-UUO. n = 6 per group.

**Figure 2:**
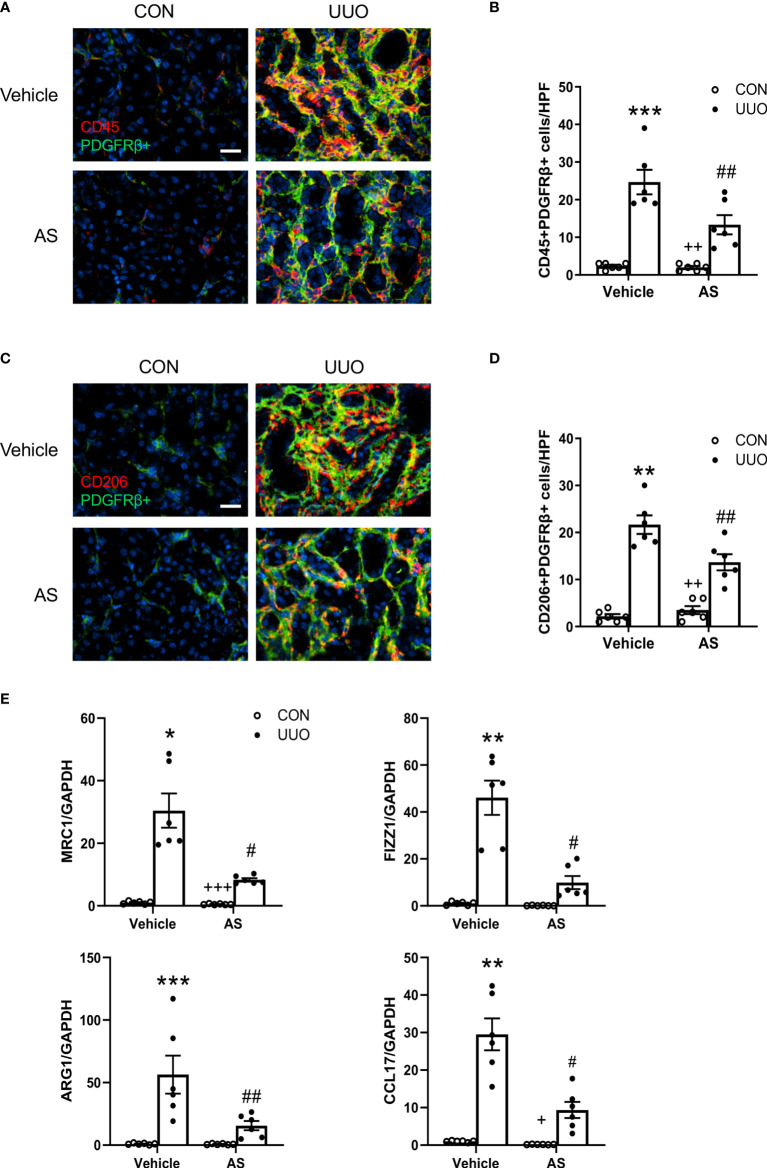
AS1517499 attenuates myeloid fibroblast accumulation and macrophage polarization in the kidney with obstructive injury. **(A)** Representative photomicrographs of kidney sections at day 10 of UUO stained for CD45 (red), PDGFR-β (green), and DAPI (blue). Scale bar, 50μm. **(B)** Quantitative analysis of CD45^+^ and PDGFR-β^+^ fibroblasts in the kidney. ***P < 0.001 *vs* Vehicle-CON; ^##^ *P <* 0.01 *vs* Vehicle-UUO; ^++^ *P <* 0.01 *vs* AS-UUO. n = 6 per group. **(C)** Representative photomicrographs of the kidney at day 10 of UUO stained for CD206 (red), PDGFR-β (green), and DAPI (blue). Scale bar, 50μm. **(D)** Quantitative analysis of CD206^+^ and PDGFR-β^+^ fibroblasts in the kidney. **P < 0.01 *vs* Vehicle-CON; ^##^ *P <* 0.01 *vs* Vehicle-UUO; ^++^ *P <* 0.01 *vs* AS-UUO. n = 6 per group. **(E)** Quantitative analysis of mRNA expression of M2 macrophage makers (MRC, FIZZ1, Arg1, CCL17 in the kidney of Vehicle or AS1517499 treated mice. ***P < 0.001, **P < 0.01, *P < 0.05 *vs* Vehicle-CON; ^##^ *P <* 0.01, ^#^ *P <* 0.05 *vs* Vehicle-UUO; ^+++^ *P <* 0.001, ^+^ *P <* 0.05 *vs* AS-UUO. n = 6 per group.

**Figure 3:**
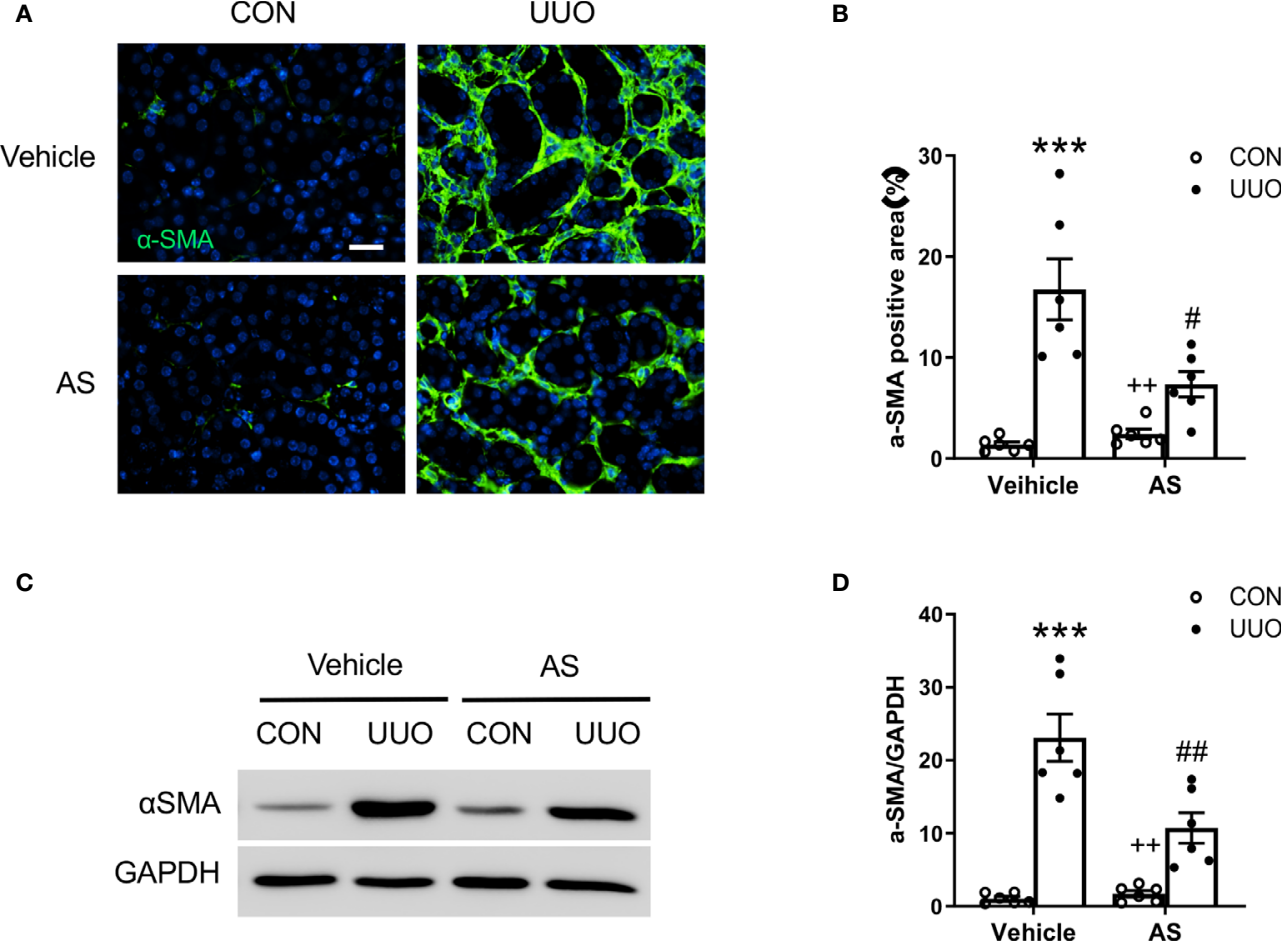
AS1517499 reduces myofibroblast formation in the kidney with obstructive injury. **(A)** Representative photomicrographs of UUO treated kidney sections stained for α-SMA (green) and counterstained with DAPI (blue). Scale bar, 50μm. **(B)** Quantitative analysis of α-SMA positive area in the kidney. ***P < 0.001 *vs* Vehicle-CON, ^#^ *P <* 0.05 *vs* Vehicle-UUO, ^++^ *P <* 0.01 *vs* AS-UUO. n = 6 per group. **(C)** Representative Western blots show α-SMA protein levels in the kidney. **(D)** Quantitative analysis of α-SMA protein expression in the kidney. ***P < 0.001 *vs* Vehicle-CON, ^##^ *P <* 0.01 *vs* Vehicle-UUO, ^++^ *P <* 0.01 *vs* AS-UUO. n = 6 per group.

**Figure 4:**
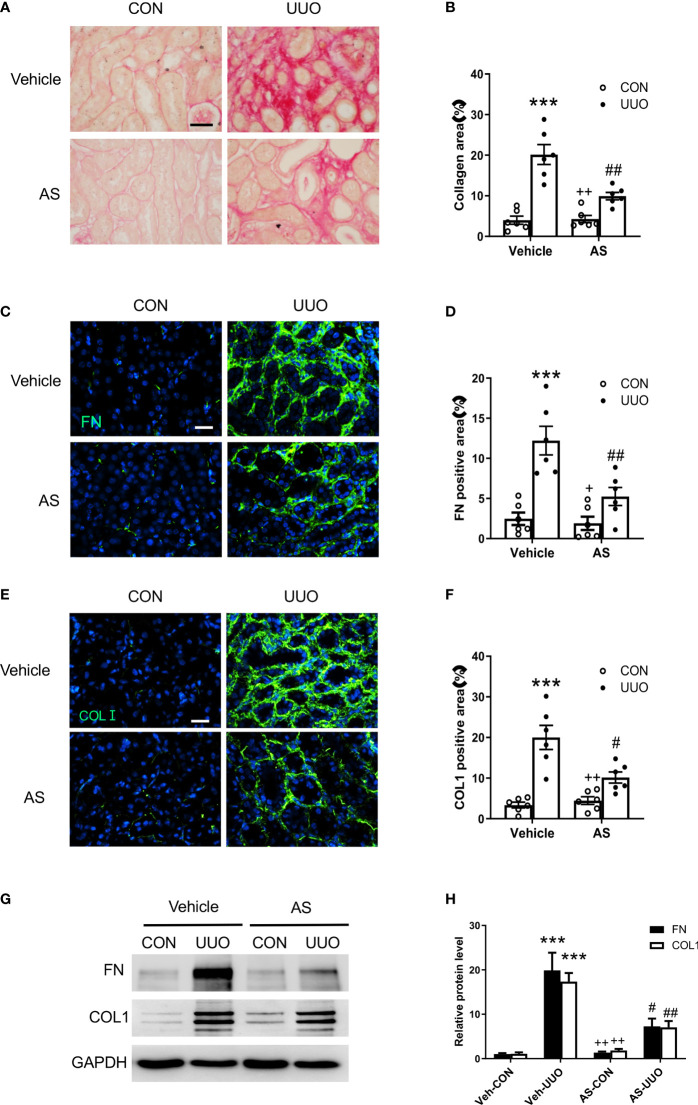
AS1517499 attenuates renal fibrosis and ECM protein production in the kidney with obstructive injury. **(A)** Representative photomicrographs of kidney sections stained with Sirius red for evaluation of total collagen deposition in the kidney. Scale bar, 50μm. **(B)** Quantitative analysis of interstitial collagen deposition in the kidney. ***P <0.001 *vs* Vehicle-CON. ^##^ *P <* 0.01 *vs* Vehicle-UUO, ^++^ *P <* 0.01 *vs* AS-UUO. n = 6 per group. **(C)** Representative photomicrographs of the kidney sections stained for fibronectin (green) and counterstained with DAPI (blue). Scale bar, 50μm. **(D)** Quantitative analysis of fibronectin-positive area in the kidney. ***P < 0.001 *vs* Vehicle-CON. ^##^ *P <* 0.01 *vs* Vehicle-UUO, ^+^ *P <* 0.05 *vs* AS-UUO. n = 6 per group. **(E)** Representative photomicrographs of the kidney sections stained for collagen I (green) and counterstained with DAPI (blue). Scale bar, 50μm. **(F)** Quantitative analysis of collagen I positive area in the kidney. ***P < 0.001 *vs* Vehicle-CON. ^#^ *P <* 0.05 *vs* Vehicle-UUO, ^++^ *P <* 0.01 *vs* AS-UUO. n = 6 per group. **(G)** Representative Western blots show protein expression of fibronectin and collagen I in the kidney. **(H)** Quantitative analysis of protein levels of fibronectin and collagen I in the kidney. ***P < 0.001 *vs* Veh-CON; ^##^ *P <* 0.01, ^#^ *P <* 0.05 *vs* Veh-UUO; ^++^ *P <* 0.01 *vs* AS-UUO. n = 6 per group.

**Figure 5:**
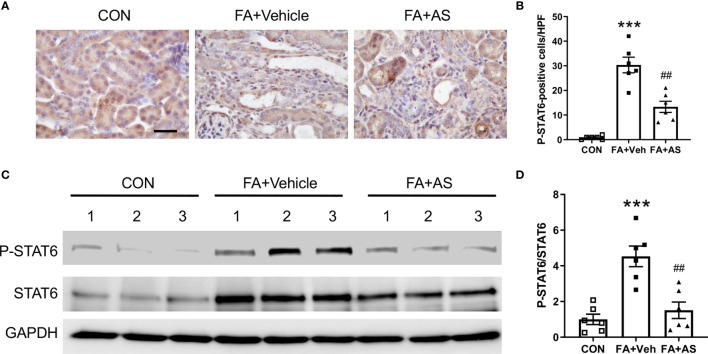
AS1517499 inhibits STAT6 activation in folic acid nephropathy. **(A)** Representative images of immunohistochemical staining of phosphorylated STAT6 in the kidney 2 weeks after vehicle or AS1517499 treatment. Scale bar, 50μm. **(B)** Quantitative analysis of phosphorylated STAT6-positive cells in the kidney 2 weeks after vehicle or AS1517499 treatment. ***P < 0.001 *vs*. CON; ^##^ *P* < 0.01 *vs* FA+Veh. n = 6 per group. **(C)** Representative Western blot of p-STAT6 and STAT6 in the kidney. **(D)** Quantitative analysis p-STAT6 protein levels in the kidney. ***P < 0.001 *vs*. CON; ^##^ *P* < 0.01 *vs* FA+Veh. n = 6 per group.

**Figure 6:**
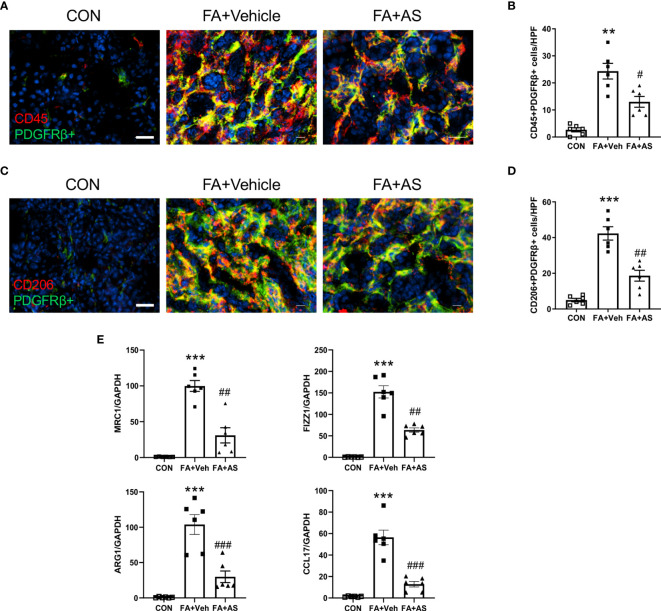
AS1517499 attenuates myeloid fibroblast accumulation and macrophage polarization in folic acid nephropathy. **(A)** Representative photomicrographs of kidney sections 2 weeks after vehicle or AS1517499 treatment stained for CD45 (red), PDGFR-β (green), and DAPI (blue). Scale bar, 50μm. **(B)** Quantitative analysis of CD45^+^ and PDGFR-β^+^ fibroblasts in the kidney. **P < 0.01 *vs* CON. ^#^ *P <* 0.05 *vs* FA+Veh. n = 6 per group. **(C)** Representative photomicrographs of kidney sections 2 weeks after vehicle or AS1517499 treatment stained for CD206 (red), PDGFR-β (green), and DAPI (blue). Scale bar, 50μm. **(D)** Quantitative analysis of CD206+ and PDGFR-β+ fibroblasts in the kidney. ***P < 0.001 *vs* CON. ^##^ *P <* 0.01 *vs* FA+Veh. n = 6 per group. **(E)** Quantitative analysis of mRNA expression of M2 macrophage makers (MRC, FIZZ1, Arg1, CCL17) in the kidney 2 weeks after vehicle or AS1517499 treatment. ***P < 0.001 *vs* CON; ^###^ *P <* 0.001 *vs* FA+Veh; ^##^ *P <* 0.01 *vs* FA+Veh. n = 6 per group.

**Figure 7:**
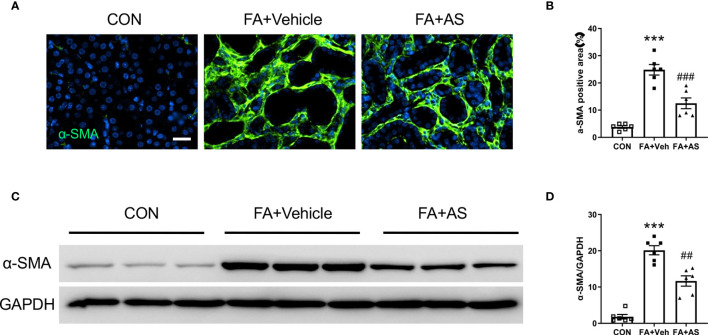
AS1517499 reduces myofibroblast formation in folic acid nephropathy. **(A)** Representative photomicrographs of kidney sections 2 weeks after vehicle or AS1517499 treatment stained for α-SMA (green) and counterstained with DAPI (blue). Scale bar, 50μm. **(B)** Quantitative analysis of α-SMA positive area in the kidney. ***P < 0.001 *vs* CON; ^###^ *P <* 0.001 *vs* FA+Veh. n = 6 per group. **(C)** Representative Western blots showing α-SMA protein levels in the kidney 2 weeks after vehicle or AS1517499 treatment. **(D)** Quantitative analysis of α-SMA protein expression in the kidney. ***P < 0.001 *vs* CON, ^##^ *P <* 0.01 *vs* FA+Veh. n = 6 per group.

**Figure 8:**
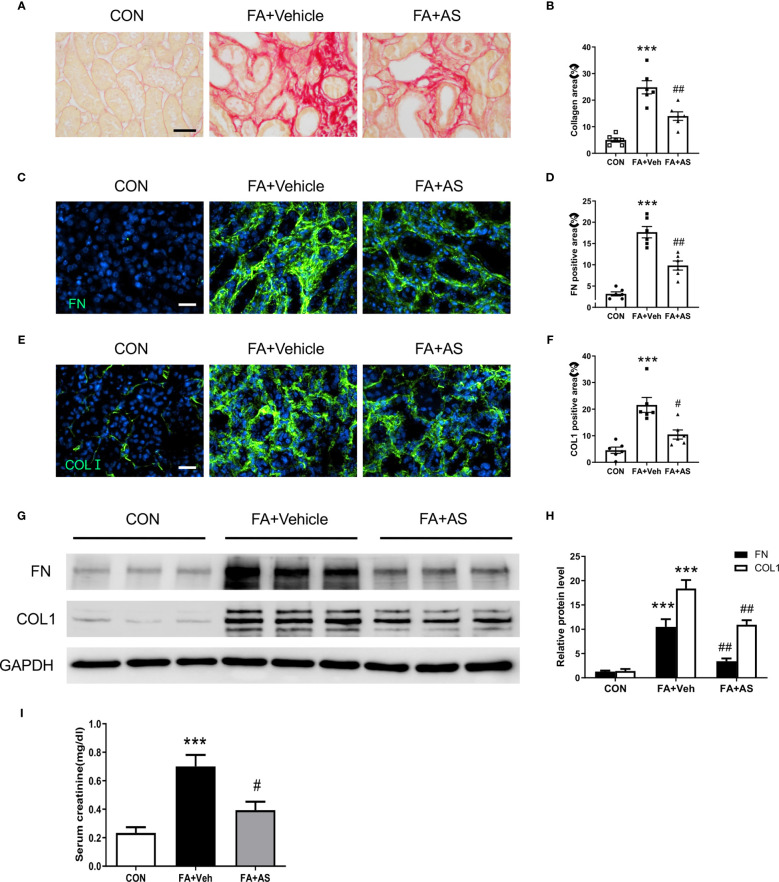
AS1517499 reduces kidney fibrosis and preserve kidney function in folic acid nephropathy. **(A)** Representative photomicrographs of kidney sections 2 weeks after vehicle or AS1517499 treatment stained with sirius red for evaluation of total collagen deposition in the kidney. Scale bar, 50μm. **(B)** Quantitative analysis of interstitial collagen deposition in the kidney. ***P < 0.001 *vs* CON, ^##^ *P <* 0.01 *vs* FA+Veh. n = 6 per group. **(C)** Representative photomicrographs of kidney sections stained for fibronectin (green) and counterstained with DAPI (blue). Scale bar, 50μm. **(D)** Quantitative analysis of fibronectin-positive area in FA treated kidneys. ***P < 0.001 *vs* CON, ^##^ *P <* 0.01 *vs* FA+Veh. n = 6 per group. **(E)** Representative photomicrographs of kidney sections stained for collagen I (green) and counterstained with DAPI (blue). Scale bar, 50μm. **(F)** Quantitative analysis of collagen I positive area in the kidney. ***P < 0.001 *vs* CON, ^#^ *P <* 0.05 *vs* FA+Veh. n = 6 per group. **(G)** Representative Western blots showing protein expression of fibronectin and collagen I in the kidney. **(H)** Quantitative analysis of protein levels of fibronectin and collagen I in the kidney. ***P < 0.001 *vs* CON; ^##^ *P <* 0.01 *vs* FA+Veh. n = 6 per group. **(I)** Effect of AS1517499 on serum creatinine. **P < 0.001 *vs* CON; ^#^ *P <* 0.05 *vs* FA+Veh. n = 6 per group.

AS1517499 was purchased from AXON Medchem LLC (Reston, VA, USA). Dimethyl sulphoxide (DMSO), 2-Mercaptoethanol and 2-Methylbutane were purchased from Sigma Chemicals Co. (St. Louis, MO, USA). Halt™ Protease and phosphatase inhibitor cocktail, RIPA Lysis and extraction buffer were obtained from Thermo Fisher Scientific (Rockford, Illinois, USA).10% Formalin was purchased from Electron Microscopy Sciences (Hatfield, PA, USA). BlockAidTM blocking solution (Invitrogen, B10710). Acetone, Hydrogen Peroxide, Acetic acid, and Xylenes were purchased from Fisher Chemical (USA). Histo-Clear II were purchased from Life Science Products (Colorado, USA). Hematoxylin, VECTASTAIN^®^ Elite ABC-HRP Kit, Peroxidase (Rabbit IgG) PK-6101, Avidin/Biotin Blocking kit, Antigen unmasking solution and ImmPACT^®^ DAB Substrate, Peroxidase (HRP) (Vector Laboratories Burlingame, CA, USA). The primary and secondary antibodies used for the Western blot were GAPDH (monoclonal, 1:5000, EMD Millipore, MAB374, RRID : AB_2107445), anti-α-SMA antibody (monoclonal, 1:500, Santa Cruz, SC-32251, RRID: AB_262054), anti-type I collagen antibody (polyclonal, 1:1000, SouthernBiotech, 1310-01, RRID: AB_2753206), anti-fibronectin antibody (polyclonal,1:1000, Sigma-Aldrich, F3648, RRID : AB_476976), phospho-STAT6 (Tyr641) antibody (polyclonal, 1:500, cell Signaling Technology, 9361, RRID : AB_331595) and STAT6 Antibody (polyclonal, 1:500, Cell Signaling Technology, 9362, RRID: AB_2271211); and secondary anti-rabbit (polyclonal,1:10000, Thermo Fisher Scientific A16035, RRID: AB_2534709), anti-goat (polyclonal,1:10000, Thermo Fisher Scientific, A16005, RRID: AB_2534679), and anti-mouse (polyclonal,1:10000, Thermo Fisher Scientific, A16017, RRID: AB_2534691), respectively. The primary antibodies used in **Figures 1**, **5** for immunohistochemistry analysis were phospho-STAT6 (Tyr641) rabbit antibody (monoclonal, Thermo Fisher Scientific Cat# 700247, RRID: AB_2532305). The primary antibodies used in **Figures 2**, **6** for immunofluorescence analysis were rat anti-CD45 (monoclonal, 1:200, BD Biosciences, 550539, RRID: AB_2174426), rat anti-CD206 (monoclonal, 1:200, Bio-Rad, MCA2235, RRID: AB_324622) and rabbit anti-PDGFRβ (polyclonal, 1:100, Santa Cruz Biotechnology, SC-432 RRID: AB_631068); the secondary antibodies Alexa Fluor 488 donkey anti-rabbit IgG (polyclonal, 1:400, Thermo Fisher Scientific, A-21206, RRID : AB_2535792) and Alexa Fluor 594 donkey anti-rat IgG (polyclonal, 1:400, Thermo Fisher Scientific, A-21209, RRID : AB_2535795). The primary antibody used in **Figures 3**, **7** for immunofluorescence analysis was mouse anti-α-SMA (monoclonal, 1:400, Santa Cruz, SC-32251, RRID: AB_262054); the secondary antibody Alexa Fluor 488-conjugated donkey anti-mouse antibody (polyclonal,1:400, Molecular Probes, A-21202, RRID: AB_141607). The primary antibodies used in **Figures 4**, **8** for immunofluorescence analysis anti-fibronectin antibody (polyclonal,1:400, Sigma-Aldrich, F3648, RRID: AB_476976), and anti-type I collagen antibody (polyclonal, 1:400, SouthernBiotech, 1310-01, RRID: AB_2753206); the secondary antibodies Alexa Fluor 488 donkey anti-rabbit IgG (polyclonal, 1:400, Thermo Fisher Scientific, A-21206, RRID: AB_2535792) and Alexa Fluor 488-conjugated donkey anti-goat antibody (polyclonal, 1: 400, Thermo Fisher Scientific, A-11055, RRID: AB_2534102).

### Animals

Wild-type (WT) C57BL/6 mice were purchased from the Jackson Laboratory. All animal experiments were conducted in accordance with national and international animal care and ethical guidelines and have been approved by the institutional animal care and use committee of the University of Connecticut Health Center. Mice were housed in groups of 3-5 littermates in a temperature-controlled environment with 12/12 h light/dark cycle and ad libitum access to food and water. Male mice at 8-10 weeks of age, weighing 20-30 grams were used in the study. In the unilateral ureteral obstruction (UUO) model, mice were anesthetized with an intraperitoneal injection of ketamine and xylazine. Through a flank incision, the left ureter was exposed and ligated at two points using fine suture material (4-0 silk) as previously described (15). In the folic acid-induced nephropathy model, WT mice were administrated intraperitoneally with folic acid (Sigma) at 250 mg/kg dissolved in 0.3 mM sodium bicarbonate (NaHCO3). Control mice were injected with equal volume of 0.3 mM NaHCO3 (16). These *in vivo* experimental models in mice replicates aspects of the human renal fibrosis (3). A randomization of animals was carried out to generate groups of equal size. The investigators responsible for data analysis were blind to the study groups.

### AS1517499 Administration

AS1517499 was dissolved in 20% DMSO and 80% normal saline. In the UUO model, mice received vehicle or AS1517499 at 10 mg/kg *via* intraperitoneal injection one hour before UUO surgery. Vehicle or AS1517499 at 10 mg/kg was administered intraperitoneally every other day following the first dose for 10 days (18).

In the folic acid-induced nephropathy model, mice received intraperitoneal injection of vehicle or AS1517499 at 10 mg/kg (18, 22) one hour before folic acid or 0.3 mM NaCO3 treatment. Vehicle or AS1517499 at 10 mg/kg was administered intraperitoneally every other day following the initial dose for 14 days.

### Western Blot Analysis

Total proteins from kidney tissues were extracted using radioimmunoprecipitation assay buffer (RIPA) buffer containing protease and phosphatase inhibitors. Equal amounts of proteins were separated on SDS-polyacrylamide gels and then electrophoretically transferred onto nitrocellulose membranes. The membranes were incubated overnight in primary antibodies (fibronectin, collagen type I, α-SMA, phosphorylated-STAT6, STAT6, or GAPDH), followed by incubation with appropriate HRP-conjugated secondary antibodies. The proteins of interest were visualized by chemiluminescence and the signal intensity was quantified using NIH ImageJ software (23).

### Immunohistochemistry

Formalin-fixed paraffin-embedded kidney tissues were sectioned at 5 μm thickness for evaluation of STAT6 phosphorylation as described (15). After deparaffinization, antigen retrieval was performed with antigen unmasking solution for 30 min, followed by quenching of endogenous peroxidase with 3% H_2_O_2_. Kidney sections were incubated with blocking buffer for 1 hour at room temperature and incubated with phospho-STAT6 antibody in a humidified chamber overnight at 4°C. The slides were washed with PBS, and then incubated with HRP-conjugated secondary antibody and ABC solution sequentially. Immunoreactivities were detected with diaminobenzidine substrate and counterstained with hematoxylin. Finally, the slides were rinsed with water, dehydrated and cover slipped. Images were obtained using a Nikon microscope equipped with a digital color camera and analyzed in a blinded manner.

### Immunofluorescence

Immunofluorescence staining was performed in frozen kidney sections. After using serum-free protein block to block nonspecific binding for 1h at room temperature, kidney sections were hybridized with primary antibodies overnight at 4°C followed by incubation with the corresponding secondary antibodies. Subsequently, slides were mounted with mounting medium containing DAPI solution. Fluorescence intensity was captured using a Nikon microscope equipped with a digital camera and analyzed in a blinded manner (24).

### Quantitative Real-Time RT-PCR

Total RNA was extracted from kidney tissues and bone marrow cells using TRIzol reagent (Invitrogen, Carlsbad, CA). Aliquots (1 μg) of total RNA were reverse transcribed using cDNA Reverse Transcription kit (Bio-Rad, Hercules, CA). Quantitative real-time PCR was performed using IQ SYBR green supermix reagent (Bio-Rad, Hercules, CA) with a Bio-Rad real-time PCR machine according to the manufacturer’s instructions. Relative mRNA expression levels of target genes were obtained by normalizing to GAPDH in each sample using the comparative Ct method (ΔΔCt) and the relative quantification was calculated as 2−ΔΔCt (25). The gene-specific primer sequences were: arginase (Arg1) forward, 5’-CTCCAAGCCAAAGTCCTTAGAG-3’,-reverse, 5’-AGGAGCTGTCATTAGGGACATC-3’;mannose receptor C-type 1 (MRC1)-forward, 5’-GGTCTATGGAACCACGGATGA-3’,-reverse, 5’-TGCCCAGTAAGGAGTACATGG-3’;found in inflammatory zone 1 (Fizz1)-forward, 5’-CCAATCCAGCTAACTATCCCTCC-3’,-reverse, 5’-ACCCAGTAGCAGTCATCCCA-3’;CCL17-forward, 5’- CGAGAGTGCTGCCTGGATTACT-3’,-reverse, 5’- GGTCTGCACAGATGAGCTTGCC-3’;GAPDH-forward, 5’-CCAATGTGTCCGTC?A3B2 ek?>GCGTGGATCT-3’,-reverse, 5’-GTTGAAGTCGCAGGAGACAACC-3’.

### Evaluation of Renal Fibrosis

Sirius red staining was performed on paraffin-imbedded kidney sections (26). After deparaffinization and hydration in distilled water, kidney sections were stained with Sirius red solution for 60 minutes to evaluate collagen fibers. Subsequently, slides were washed with acetic acid solution and absolute alcohol sequentially. The Sirius red-stained kidney sections were visualized using a microscope equipped with a digital camera (Nikon, Melville, NY) and quantitative evaluation was performed in a blinded fashion using NIS-Elements Br 3.0 software (9).

### Kidney Function Assay

Serum creatinine was measured using a commercially available kit (BioAssay Systems, Hayward, CA) as reported (27, 28).

### Statistical Analysis

All values were expressed as mean ± SEM. Multiple comparisons were performed by Analysis of variance (ANOVA) followed by the Bonferroni procedure for comparison of means. A p-value of less than 0.05 was considered statistically significant.

## Results

### AS1517499 Inhibits STAT6 Activation in the Kidney With Obstructive Injury

We have reported that JAK3/STAT6 signaling is activated in interstitial cells of fibrotic kidneys following obstructive injury or folic acid administration (15, 16). To evaluate whether STAT6 can serve as a therapeutic target for the treatment of renal fibrosis, wild type mice were subjected to obstructive injury and then treated with vehicle or AS1517499, a STAT6-specific inhibitor, every two days for 10 days. Immunohistochemical staining and Western blot analysis were performed to examine STAT6 activation in the kidney. Immunohistochemical staining revealed that positive phosphor-STAT6 staining was mainly detected in the interstitial cells of the kidney with obstructive nephropathy, which was reduced by AS1517499 treatment (**Figures 1A, B**). Consistent with these findings, Western blot analysis showed level of phospho-STAT6 was inhibited in the kidney with obstructive nephropathy following AS1517499 treatment (**Figures 1C, D**). These data indicate that the inhibition of STAT6 with AS1517499 prevents STAT6 activation in the kidney with obstructive nephropathy.

### AS1517499 Impairs Myeloid Fibroblast Accumulation in Obstructive Nephropathy

Accumulating evidence indicates that the recruitment of myeloid fibroblasts contributes significantly to the pathogenesis of renal fibrosis. To investigate whether AS1517499 affects myeloid fibroblasts accumulation, kidney sections were stained for the hematopoietic marker CD45 and mesenchymal marker PDGFR-β. The number of CD45 and PDGFR-β dual positive cells was significantly enhanced in the obstructed kidneys, whereas AS1517499 treatment markedly reduced CD45 and PDGFR-β dual positive cells (**Figures 2A, B**). These data suggest that STAT6 inhibitor AS1517499 ameliorates myeloid fibroblasts accumulation in the kidney after obstructive injury.

### AS1517499 Attenuates M2 Macrophage Polarization in Obstructive Nephropathy

The transformation of monocytes into bone marrow derived fibroblasts is mediated by M2 macrophage polarization. STAT6 plays a key role in M2 macrophage polarization. Therefore, we examined whether the inhibition of STAT6 with AS1517499 mediates macrophage polarization. Kidney sections were stained for CD206 (M2 macrophage marker) and PDGFR-β. CD206 and PDGFR-β dual positive cells were markedly increased in the kidney of mice with obstructive injury. In contrast, AS1517499 significantly reduced the number of CD206+ and PDGFR-β+ cells in kidney with obstructive nephropathy (**Figures 2C, D**). These data indicate that AS1517499 significantly attenuates the transition of monocytes into myeloid fibroblasts and reduces M2 macrophage polarization.

To further evaluate the effect of STAT6 inhibitor AS1517499 on M2 macrophage polarization, real-time RT-PCR was performed to detect mRNA expressions of M2 macrophage markers. AS1517499 treatment suppressed mRNA expression abundance of Arg1, MRC1, Fizz1, and CCL17 in obstructed kidneys (**Figure 2E**). These data indicate that STAT6 signaling promotes M2 macrophage polarization while pharmacological inhibition of STAT6 with AS1517499 prevents M2 macrophage differentiation in the damaged kidneys.

### AS1517499 Inhibits Myofibroblast Formation in Obstructive Nephropathy

Activated fibroblasts, which are termed myofibroblasts (29), are responsible for the production and accumulation of large amounts of interstitial ECM components in the kidney (30). We next examined the effect of AS1517499 on myofibroblast transformation in the kidney by assessing α-smooth muscle actin (α-SMA) expression in the kidney. Immunohistochemical staining demonstrated that AS1517499 treatment suppressed the number of α-SMA positive myofibroblasts in the kidney with obstructive nephropathy (**Figures 3A, B**). Western blot analysis showed that AS1517499 treatment reduced the expression level of α-SMA protein in the obstructed kidney compared with vehicle-treated UUO group (**Figures 3C, D**).

### AS1517499 Ameliorates Renal Fibrosis and ECM Protein Production in Obstructive Nephropathy

To evaluate the effect of AS1517499 on renal fibrosis, Sirius Red staining was performed on kidney sections. Sirius red staining showed significant interstitial collagen deposition in the obstructed kidney, whereas AS1517499 treatment significantly attenuated interstitial collagen deposition in the kidney subjected to UUO injury (**Figures 4A, B**).

To investigate whether AS1517499 could affect the production of ECM proteins (collagen I and fibronectin) in the kidney with obstructive nephropathy, we performed immunofluorescence staining and Western blot analysis. Immunofluorescence staining and Western blot analysis revealed the levels of fibronectin and collagen I were markedly increased in the obstructed kidney, whereas AS1517499 treatment significantly attenuated the protein levels of collagen I and fibronectin in the obstructed kidney (**Figures 4C–H**).

### AS1517499 Inhibits STAT6 Activation in Folic Acid Nephropathy

Folic acid nephropathy is another commonly used murine model of renal fibrosis. Therefore, we examined whether AS1517499 can inhibit STAT6 activation and pathogenesis of renal fibrosis in folic acid nephropathy. Kidney sections were stained for phospho-STAT6. STAT6 phosphorylation was significantly induced in interstitial cells of the kidney with FA nephropathy, whereas AS1517499 treatment markedly inhibited the expression of phospho-STAT6 (**Figures 5A, B**). These findings were further confirmed by Western blot analysis (**Figures 5C, D**). These data indicate that STAT6 is activated in the interstitial cells of the damaged kidney with folic acid nephropathy, which is significantly inhibited following AS1517499 treatment.

### AS1517499 Suppresses Myeloid Fibroblast Accumulation in Folic Acid Nephropathy

To examine the role of AS1517499 in the myeloid fibroblast accumulation in the kidney with folic acid nephropathy, we performed double immunofluorescence staining for CD45 and PDGFR-β. The number of CD45 and PDGFR-β dual positive cells increased in the kidney with folic acid nephropathy. AS1517499 considerably suppressed the number of CD45 and PDGFR-β dual positive cells in the kidney with folic acid nephropathy (**Figures 6A, B**).

### AS1517499 Attenuates M2 Macrophage Polarization in Folic Acid Nephropathy

We then examined whether the pharmacological inhibition of STAT6 with AS1517499 regulates M2 macrophage polarization in FA nephropathy. As confirmed by immunofluorescence staining analysis, treatment of AS1517499 significantly inhibited the number of CD206 and PDGFR-β dual positive cells compared with vehicle treated mice with FA administration (**Figures 6C, D**). In addition, M2 macrophage markers, Arg1, MRC1, Fizz1, and CCL17 were induced in folic acid nephropathy. These M2 macrophage markers were reduced following AS1517499 treatment (**Figure 6E**). These data indicate that the pharmacological inhibition of STAT6 with AS1517499 attenuates M2 macrophage polarization in the kidney with FA nephropathy.

### AS1517499 Attenuates Myofibroblast Formation in Folic Acid Nephropathy

To determine whether AS1517499 affects myofibroblast transformation, α-SMA expression was examined in kidney sections and by western blot analysis. Consistent with our findings in the UUO model, α-SMA positive myofibroblasts and the expression of α-SMA protein was enhanced in the kidney after treatment with FA compared with vehicle treated mice. Moreover, co-administration of FA with AS1517499 significantly reduced α-SMA positive cells and α-SMA protein level in the kidney compared with FA co-administrated vehicle group (**Figures 7A–D**). These results demonstrate that AS1517499 inhibits myofibroblast formation in the kidney with folic acid nephropathy.

### AS1517499 Ameliorates Renal Fibrosis and Preserves Renal Function in Folic Acid Nephropathy

To evaluate the effect of AS1517499 on renal fibrosis, picrosirius red staining was performed on kidney sections. FA-treated mice had markedly elevated levels of collagen deposition, whereas AS1517499 treatment significantly reduced the amounts of collagen deposition in the kidney (**Figures 8A, B**). Additionally, immunofluorescence staining and Western blot analysis further demonstrated that AS1517499 significantly inhibits the expression of ECM proteins (fibronectin and collagen I) in the kidney with folic acid nephropathy (**Figures 8C–H**). These data suggest that AS1517499 inhibits ECM production in the kidney and the development of renal fibrosis in folic acid nephropathy.

To assess the effect of STAT6 deficiency on kidney function in folic acid nephropathy, serum creatinine was measured. Folic acid treated mice displayed a significant elevation of serum creatinine (**Figure 8I**). In contrast, mice treated with AS1517499 exhibited much lower serum creatinine in folic acid nephropathy. These results indicate that inhibition of STAT6 with AS1517499 protects the kidney from folic acid nephropathy.

## Discussion

Renal fibrosis is a key pathological feature of chronic kidney disease. At present, there are no effective treatments or specific drugs for preventing and/or reversing renal fibrosis progression. Studies have demonstrated that a major source of myofibroblasts originates from bone marrow-derived fibroblasts, which is derived from monocyte subpopulations through the differentiation of monocytes into fibroblasts (9, 23, 30, 31). However, the molecular mechanisms underlying monocytes-to-fibroblast transition in the injured kidney are not fully elucidated. In the present study, we examined the effect of a STAT6-specific inhibitor, AS1517499, on monocyte-to-fibroblast transition, M2 macrophage polarization, and development of renal fibrosis in two murine models of chronic kidney disease induced by ureteral obstruction or folic acid. Our results show that treatment with AS1517499 in mice with obstructive nephropathy or FA nephropathy suppresses myeloid fibroblast accumulation, decreases M2 macrophage polarization and myofibroblast transformation, reduces ECM protein production and collagen deposition in the kidney, and prevents kidney dysfunction.

We have showed that the IL4Rα/JAK3/STAT6 signaling pathway play an important role in the activation of myeloid fibroblast and the development of renal fibrosis (15). Genetic disruption of IL4Rα, pharmacological inhibition of JAK3, or genetic deletion of STAT6 reduces myeloid fibroblast accumulation and activation in the kidney in response to obstructive injury or folic acid injury (15, 16). AS1517499 is a potent and selective STAT6 inhibitor with an IC50 of 21 nM (21). Chiba and colleagues report that AS1517499 inhibits antigen-induced hyperresponsiveness in a murine model of asthma (22). In the present study, we examine the effect of pharmacological inhibition of STAT6 with AS1517499 on myeloid fibroblast activation in experimental models of renal fibrosis induced by ureteral obstruction or folic acid. Our results reveal that STAT6 is phosphorylated in the kidney in response to obstructive injury or folic acid injury. The level of STAT6 phosphorylation in the kidney is markedly inhibited by AS1517499 administration. These data indicate that AS1517499 inhibits STAT6 activation in the kidney with injury. Of note, the total STAT6 levels in the kidney are increased following ureteral obstruction or folic acid administration, which is somewhat reduced after AS1517499 treatment. This may be related to STAT6 protein stability/degradation or decreased expression of STAT6 as consequences of negative feedback regulation caused by inactivation of STAT6 signaling. Further studies are needed to elucidate the exact mechanisms. We observed that AS1517499 treatment inhibits myeloid fibroblast accumulation and activation in the kidney with injury. These results support an important role of STAT6 in the activation of myeloid fibroblasts in the kidney.

Th2 cytokines IL-4 and IL-13 stimulate STAT6 phosphorylation, which is involved in M2 macrophage polarization (15). M2 macrophages are characterized by expressing Arg1, MRC1, Fizz1, CCL17 (32). M2 macrophages have reported to promote tissue fibrosis (33–35). However, how M2 macrophages promote tissue fibrosis is not clearly understood. We have previously demonstrated that myeloid fibroblasts are derived from monocytes through M2 macrophage polarization (15, 24). Recently, Binnemars-Postma and colleagues report that inhibition of STAT6 with AS1517499 attenuates tumor associated macrophages into M2 phenotypes and reduces tumor growth and metastasis (36). In the present study, we show that treatment with AS1517499 inhibits the number of CD206 and PDGFR-β dual positive cells in the injured kidney and reduces the mRNA expression levels of M2 macrophage markers, Arg1, MRC1, Fizz1, CCL17. These data indicate that STAT6 inhibitor A1517499 suppresses monocyte-to-fibroblast transition and M2 macrophage polarization in the injured kidney during the development of renal fibrosis.

Renal fibrosis is characterized by production and deposition of ECM proteins leading to destruction of renal parenchyma and loss of kidney function (2). In the present study, we have shown that inhibition of STAT6 with AS1517499 reduces the production of ECM proteins – fibronectin and collagen I as well as collagen deposition in the kidney in two experimental models of obstructive nephropathy and folic acid nephropathy. Furthermore, pharmacological inhibition of STAT6 with AS1517499 protects the kidney from folic acid-induced kidney dysfunction. These results indicate that AS1517499 can inhibit the development of kidney fibrosis and dysfunction.

In summary, our findings indicate that STAT6 is activated in injured kidneys, leading to the accumulation and activation of myeloid fibroblasts, M2 macrophage polarization, and development of kidney fibrosis and dysfunction. Pharmacological inhibition of STAT6 with AS1517499 suppresses myeloid fibroblasts accumulation and activation, reduces M2 macrophage polarization, decreases ECM protein production, and attenuates the development of kidney fibrosis and dysfunction. These results indicate that targeting STAT6 with AS1517499 is a promising therapeutic approach for the treatment of chronic kidney disease.

## Data Availability Statement

The raw data supporting the conclusions of this article will be made available by the authors, without undue reservation.

## Ethics Statement

The animal study was reviewed and approved by IACUC at UConn Health.

## Author Contributions

BJ and YW conceived and designed the experiments. BJ performed the experiments and analyzed the data. BJ, CA, MT, HD, PW, DZ, and YW interpreted the data. BJ, MT, and YW wrote the manuscript. All authors contributed to the article and approved the submitted version.

## Funding

This work was supported by grants from the NIH/NIDDK (R01DK95835), the U.S. Department of Veterans Affairs (I01BX02650) and the Dialysis Clinic Inc (2019–03) to YW.

## Author Disclaimer

The contents of the article do not represent the views of the U.S. Department of Veterans Affairs or the United States government.

## Conflict of Interest

The authors declare that the research was conducted in the absence of any commercial or financial relationships that could be construed as a potential conflict of interest.

## Publisher’s Note

All claims expressed in this article are solely those of the authors and do not necessarily represent those of their affiliated organizations, or those of the publisher, the editors and the reviewers. Any product that may be evaluated in this article, or claim that may be made by its manufacturer, is not guaranteed or endorsed by the publisher.

## References

[1] Levey AS Atkins R Coresh J Cohen EP Collins AJ Eckardt KU. Chronic Kidney Disease as a Global Public Health Problem: Approaches and Initiatives - A Position Statement From Kidney Disease Improving Global Outcomes. Kidney Int (2007) 72:247–59. 10.1038/sj.ki.5002343 17568785

[2] Liu Y. Cellular and Molecular Mechanisms of Renal Fibrosis. Nat Rev Nephrol (2011) 7:684–96. 10.1038/nrneph.2011.149 PMC4520424 22009250

[3] Nogueira A Pires MJ Oliveira PA. Pathophysiological Mechanisms of Renal Fibrosis: A Review of Animal Models and Therapeutic Strategies. In Vivo (2017) 31:1–22. 10.21873/invivo.11019 28064215 PMC5354133

[4] An C Jia L Wen J Wang Y. Targeting Bone Marrow-Derived Fibroblasts for Renal Fibrosis. Adv Exp Med Biol (2019) 1165:305–22. 10.1007/978-981-13-8871-2_14 31399971

[5] Xu J Kisseleva T. Bone Marrow-Derived Fibrocytes Contribute to Liver Fibrosis. Exp Biol Med (Maywood) (2015) 240:691–700. 10.1177/1535370215584933 25966982 PMC4866973

[6] Xu J Cong M Park TJ Scholten D Brenner DA Kisseleva T. Contribution of Bone Marrow-Derived Fibrocytes to Liver Fibrosis. Hepatobil Surg Nutr (2015) 4:34–47. 10.3978/j.issn.2304-3881.2015.01.01 PMC4318956 25713803

[7] Sakai N Wada T. T Helper 2 Cytokine Signaling in Bone Marrow-Derived Fibroblasts: A Target for Renal Fibrosis. J Am Soc Nephrol (2015) 26:2896–8. 10.1681/ASN.2015040469 PMC4657850 26032812

[8] Reich B Schmidbauer K Rodriguez Gomez M Johannes Hermann F Gobel N Bruhl H. Fibrocytes Develop Outside the Kidney But Contribute to Renal Fibrosis in a Mouse Model. Kidney Int (2013) 84:78–89. 10.1038/ki.2013.84 23486523

[9] Chen G Lin SC Chen J He L Dong F Xu J. CXCL16 Recruits Bone Marrow-Derived Fibroblast Precursors in Renal Fibrosis. J Am Soc Nephrol (2011) 22:1876–86. 10.1681/ASN.2010080881 PMC3187185 21816936

[10] Jang HS Kim JI Jung KJ Kim J Han KH Park KM. Bone Marrow-Derived Cells Play a Major Role in Kidney Fibrosis *via* Proliferation and Differentiation in the Infiltrated Site. Biochim Biophys Acta (2013) 1832:817–25. 10.1016/j.bbadis.2013.02.016 23466592

[11] Jang HS Kim JI Han SJ Park KM. Recruitment and Subsequent Proliferation of Bone Marrow-Derived Cells in the Postischemic Kidney Are Important to the Progression of Fibrosis. Am J Physiol Renal Physiol (2014) 306:F1451–61. 10.1152/ajprenal.00017.2014 24740786

[12] Jiang Y Wang Y Ma P An D Zhao J Liang S. Myeloid-Specific Targeting of Notch Ameliorates Murine Renal Fibrosis *via* Reduced Infiltration and Activation of Bone Marrow-Derived Macrophage. Protein Cell (2019) 10:196–210. 10.1007/s13238-018-0527-6 29644573 PMC6338623

[13] Friedenstein AJ Deriglasova UF Kulagina NN Panasuk AF Rudakowa SF Luria EA. Precursors for Fibroblasts in Different Populations of Hematopoietic Cells as Detected by the *In Vitro* Colony Assay Method. Exp Hematol (1974) 2:83–92. 4455512

[14] Ichim TE O’Heeron P Kesari S. Fibroblasts as a Practical Alternative to Mesenchymal Stem Cells. J Transl Med (2018) 16:212. 10.1186/s12967-018-1536-1 30053821 PMC6064181

[15] Yan J Zhang Z Yang J Mitch WE Wang Y. JAK3/STAT6 Stimulates Bone Marrow-Derived Fibroblast Activation in Renal Fibrosis. J Am Soc Nephrol (2015) 26:3060–71. 10.1681/ASN.2014070717 PMC4657828 26032813

[16] Liang H Zhang Z Yan J Wang Y Hu Z Mitch WE. The IL-4 Receptor Alpha Has a Critical Role in Bone Marrow-Derived Fibroblast Activation and Renal Fibrosis. Kidney Int (2017) 92:1433–43. 10.1016/j.kint.2017.04.021 PMC5696054 28739140

[17] Walford HH Doherty TA. STAT6 and Lung Inflammation. JAKSTAT (2013) 2:e25301. 10.4161/jkst.25301 24416647 PMC3876430

[18] Kim MJ Lee YJ Yoon YS Lim JH Park EM Chong YH. A STAT6 Inhibitor AS1517499 Reduces Preventive Effects of Apoptotic Cell Instillation on Bleomycin-Induced Lung Fibrosis by Suppressing PPARgamma. Cell Physiol Biochem (2018) 45:1863–77. 10.1159/000487877 29510393

[19] Du P Ma Q Zhu ZD Li G Wang Y Li QQ. Mechanism of Corilagin Interference With IL-13/STAT6 Signaling Pathways in Hepatic Alternative Activation Macrophages in Schistosomiasis-Induced Liver Fibrosis in Mouse Model. Eur J Pharmacol (2016) 793:119–26. 10.1016/j.ejphar.2016.11.018 27845069

[20] Yukawa K Kishino M Goda M Liang XM Kimura A Tanaka T. STAT6 Deficiency Inhibits Tubulointerstitial Fibrosis in Obstructive Nephropathy. Int J Mol Med (2005) 15:225–30. 10.3892/ijmm.15.2.225 15647835

[21] Nagashima S Yokota M Nakai E Kuromitsu S Ohga K Takeuchi M. Synthesis and Evaluation of 2-{[2-(4-Hydroxyphenyl)-Ethyl]Amino}Pyrimidine-5-Carboxamide Derivatives as Novel STAT6 Inhibitors. Bioorg Med Chem (2007) 15:1044–55. 10.1016/j.bmc.2006.10.015 17071093

[22] Chiba Y Todoroki M Nishida Y Tanabe M Misawa M. A Novel STAT6 Inhibitor AS1517499 Ameliorates Antigen-Induced Bronchial Hypercontractility in Mice. Am J Respir Cell Mol Biol (2009) 41:516–24. 10.1165/rcmb.2008-0163OC 19202006

[23] Xia Y Yan J Jin X Entman ML Wang Y. The Chemokine Receptor CXCR6 Contributes to Recruitment of Bone Marrow-Derived Fibroblast Precursors in Renal Fibrosis. Kidney Int (2014) 86:327–37. 10.1038/ki.2014.64 PMC4117803 24646857

[24] Yang J Lin SC Chen G He L Hu Z Chan L. Adiponectin Promotes Monocyte-to-Fibroblast Transition in Renal Fibrosis. J Am Soc Nephrol (2013) 24:1644–59. 10.1681/ASN.2013030217 PMC3785282 23833260

[25] An C Wen J Hu Z Mitch WE Wang Y. Phosphoinositide 3-Kinase Gamma Deficiency Attenuates Kidney Injury and Fibrosis in Angiotensin II-Induced Hypertension. Nephrol Dial Transplant (2020) 35:1491–500. 10.1093/ndt/gfaa062 PMC7778344 32500132

[26] Wang Y Jia L Hu Z Entman ML Mitch WE Wang Y. AMP-Activated Protein Kinase/Myocardin-Related Transcription Factor-A Signaling Regulates Fibroblast Activation and Renal Fibrosis. Kidney Int (2018) 93:81–94. 10.1016/j.kint.2017.04.033 28739141 PMC5750062

[27] Jin X Chen J Hu Z Chan L Wang Y. Genetic Deficiency of Adiponectin Protects Against Acute Kidney Injury. Kidney Int (2013) 83:604–14. 10.1038/ki.2012.408 PMC3612389 23302722

[28] Zhou J Jia L Hu Z Wang Y. Pharmacological Inhibition of PTEN Aggravates Acute Kidney Injury. Sci Rep (2017) 7:9503. 10.1038/s41598-017-10336-8 28842716 PMC5572703

[29] Hinz B Phan SH Thannickal VJ Galli A Bochaton-Piallat ML Gabbiani G. The Myofibroblast: One Function, Multiple Origins. Am J Pathol (2007) 170:1807–16. 10.2353/ajpath.2007.070112 PMC1899462 17525249

[30] Lebleu VS Taduri G O’Connell J Teng Y Cooke VG Woda C. Origin and Function of Myofibroblasts in Kidney Fibrosis. Nat Med (2013) 19:1047–53. 10.1038/nm.3218 PMC4067127 23817022

[31] Niedermeier M Reich B Rodriguez Gomez M Denzel A Schmidbauer K Gobel N. CD4+ T Cells Control the Differentiation of Gr1+ Monocytes Into Fibrocytes. Proc Natl Acad Sci USA (2009) 106:17892–7. 10.1073/pnas.0906070106 PMC2764893 19815530

[32] Murray PJ. Macrophage Polarization. Annu Rev Physiol (2017) 79:541–66. 10.1146/annurev-physiol-022516-034339 27813830

[33] Anders HJ Ryu M. Renal Microenvironments and Macrophage Phenotypes Determine Progression or Resolution of Renal Inflammation and Fibrosis. Kidney Int (2011) 80:915–25. 10.1038/ki.2011.217 21814171

[34] Pechkovsky DV Prasse A Kollert F Engel KM Dentler J Luttmann W. Alternatively Activated Alveolar Macrophages in Pulmonary Fibrosis-Mediator Production and Intracellular Signal Transduction. Clin Immunol (2010) 137:89–101. 10.1016/j.clim.2010.06.017 20674506

[35] Cao Q Wang Y Harris DC. Macrophage Heterogeneity, Phenotypes, and Roles in Renal Fibrosis. Kidney Int Suppl (2011) (2014) 4:16–9. 10.1038/kisup.2014.4 PMC4536959 26312145

[36] Binnemars-Postma K Bansal R Storm G Prakash J. Targeting the Stat6 Pathway in Tumor-Associated Macrophages Reduces Tumor Growth and Metastatic Niche Formation in Breast Cancer. FASEB J (2018) 32:969–78. 10.1096/fj.201700629R 29066614

